# Controllable Thermal Rectification Realized in Binary Phase Change Composites

**DOI:** 10.1038/srep08884

**Published:** 2015-03-09

**Authors:** Renjie Chen, Yalong Cui, He Tian, Ruimin Yao, Zhenpu Liu, Yi Shu, Cheng Li, Yi Yang, Tianling Ren, Gang Zhang, Ruqiang Zou

**Affiliations:** 1 Department of Materials Science and Engineering, College of Engineering, Peking University, Beijing 100871, China; 2 Institute of Microelectronics, Tsinghua University, Beijing 100084, China; 3 Tsinghua National Laboratory for Information Science and Technology (TNList), Tsinghua University, Beijing 100084, China; 4 Institute of High Performance Computing, Singapore 138632, Singapore

## Abstract

Phase transition is a natural phenomenon happened around our daily life, represented by the process from ice to water. While melting and solidifying at a certain temperature, a high heat of fusion is accompanied, classified as the latent heat. Phase change material (PCM) has been widely applied to store and release large amount of energy attributed to the distinctive thermal behavior. Here, with the help of nanoporous materials, we introduce a general strategy to achieve the binary eicosane/PEG4000 stuffed reduced graphene oxide aerogels, which has two ends with different melting points. It's successfully demonstrated this binary PCM composites exhibits thermal rectification characteristic. Partial phase transitions within porous networks instantaneously result in one end of the thermal conductivity saltation at a critical temperature, and therefore switch on or off the thermal rectification with the coefficient up to 1.23. This value can be further raised by adjusting the loading content of PCM. The uniqueness of this device lies in its performance as a normal thermal conductor at low temperature, only exhibiting rectification phenomenon when temperature is higher than a critical value. The stated technology has broad applications for thermal energy control in macroscopic scale such as energy-efficiency building or nanodevice thermal management.

The conduction of heat is one of the fundamental energy transport mechanisms in nature^1^,^2^. Usually, more attentions are focused on the control of heat energy transfer^3^,^4^,^5^ through phonon engineering^6^,^7^ and nanostructuring^8^,^9^,^10^. Recently, phononics (thermal) devices, which can control the flow direction of heat energy, have grown considerably in importance for the understanding of how heat energy is transported, distributed and converted from fundamental science to applied research field^11^,^12^,^13^,^14^,^15^. In addition to future information processing as thermal computers, the phononics devices have extensive applications for heat energy management as well^16^,^17^. Similar to electric counterpart, the thermal rectifier is one of the most fundamental phononic components. The first solid-state thermal rectifier based on a single carbon nanotube has been reported experimentally^18^, just four years after the simulation result reported. The underlying mechanism of the rectification phenomenon can be understood by the match/mismatch of the power spectra between the two ends of the device^19^. Although various nanostructures have been theoretically predicted that they can present thermal rectification characteristic according to the mismatch of the phonon spectra since mass or geometric asymmetry^20^,^21^,^22^,^23^,^24^,^25^, they usually confronted with the design and fabrication challenges. However, the design of these reported nanoscale and macroscopic thermal rectifiers depends on the symmetry of whole system, and the rectification characteristic need be fixed after fabrication. Recently, thermal rectifiers in macroscopic scale built from perovskite cobalt oxides^24^,^25^ and reduced graphene oxide (rGO) were realized^26^. Distinct from the rGO thermal rectifier in the light of the asymmetry of rGO paper, the perovskite oxide thermal rectifier was demonstrated by the “jump” of thermal conductivity originated in the structural phase transition with the rectification coefficient of 1.14 in the presence of a small temperature difference of 2 K. This work has a significant contribution to control heat current in electronic devices. On the whole, these achievements have raised the exciting prospect that the realization of phononics devices is technologically feasible, and open the possibility for smart thermal energy management in the future.

In contrast to the thermal rectifiers mentioned above, we conceive a new mechanism to realize the thermal rectification in terms of the thermal conductivity saltation through the solid-liquid phase transition. Generally, practical applications of solid-liquid phase change materials (PCMs) have been hindered by a number of challenges including their leakage and considerable volume expansion during phase transition. One of the most efficient ways to solve these disadvantages is using nano-confinement technology, which not only can effectively prevent liquid leakage, but also can realize the functionality of PCMs, *i.e.* electro-to-heat and photo-to-thermal conversion^27^,^28^,^29^,^30^,^31^. Carbon nanotube sponge^29^ or array^30^, graphene aerogel^31^ can be selected as promising nanoporous skeleton candidates. Moreover, the structure of porous framework would influence on the phase transition behavior, such as latent heat and phase transition temperature. In this paper, we introduce a general strategy to manufacture a new thermal rectifier built from binary solid-liquid PCMs, which was stuffed in the two ends of the same nanoporous scaffold. The difference in thermal conductivity of two ends of the scaffold, derived from the phase transition of PCMs, acts as the vital role to regulate thermal performances. Using binary eicosane/PEG4000-stuffed (PEG4000 = polyethylene glycol 4000) rGO aerogels as an example, we have achieved temperature-responded switch-on or switch-off status of the thermal rectifier, with a rectification coefficient up to 1.23. The generality of the approach is also demonstrated in multicomponent phase change composites with different temperature gradients. The thermal rectification can be modulated through setting different input temperature or tuning the loading of various PCMs. In addition to as a thermal rectifier, this composite can be applied in thermal memory as well^32^.

## Results

### Device design

We use binary eicosane/PEG4000 stuffed rGO aerogels as an example. The phase transition temperatures of eicosane and PEG4000 are around 35 and 60°C. The deformable rGO aerogel scaffold with the size of 25.0 × 6.0 × 0.8 mm^3^ was prepared by hydrothermal method and supercritical dry subsequently according to the references 33, 34 (Supplemental Information). The composite fabrication simply involves solvent-assisted infiltration of PCMs into the inner free space of rGO aerogel (Figure S1). Normally, the surface energy of porous skeleton and surface tension of PCMs are two key factors to determine the infiltration process. In this case, rGO aerogel shows much lower surface energy than the molten PCMs, and its small pores are beneficial to rapidly adsorb PCM droplets. Meanwhile, some air gaps or pockets are expected to form between the PCMs and the pore surface, and these air gaps can develop a pressure^35^. The pressure difference between the liquid PCMs and air gaps is known as the capillary pressure, which can try to push against the surface tension of the PCMs.

**Figure 1:**
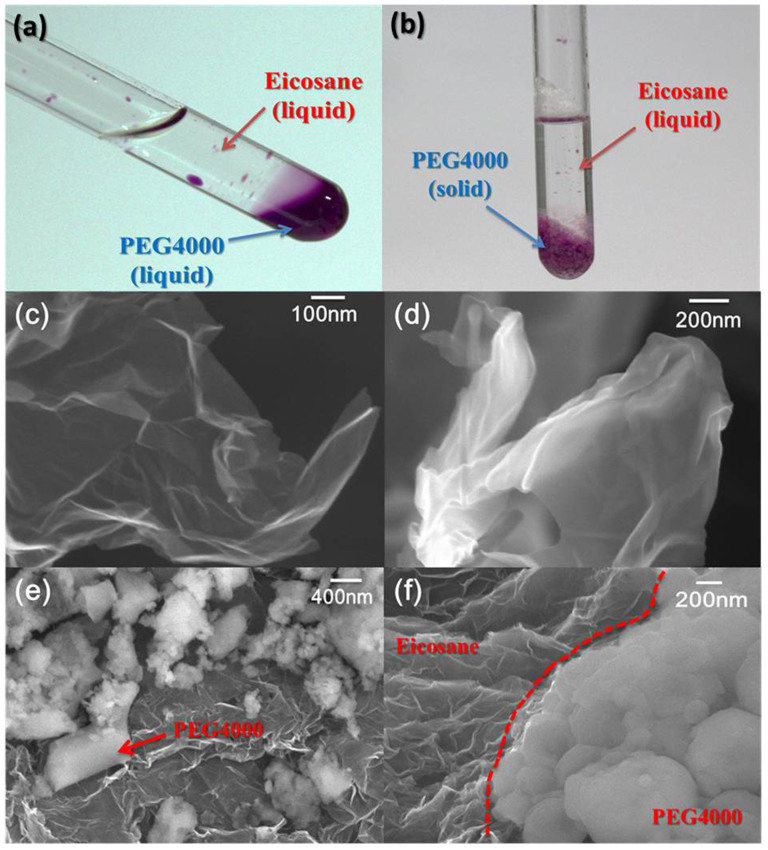
SEM images of composites. (a) Photo of liquid state PCMs. (b) Photo of PCMs at 35°C. (c) The rGO sheets of aerogel. (d) The rGO aerogel sheets wrapped by eicosane. (e) PEG4000 particles separated in the rGO sheets. (f) The boundary of the eicosane and PEG4000 in the rectifier.

The resulting composite, denoted as PCMs@rGO, consists of a three-dimensional (3D) rGO scaffold in which the inner intersheets and pores are completely or partially filled by eicosane and PEG4000. The two PCMs with different phase transition temperatures and enthalpies in two ends are immiscible in liquid state (Figure 1a and 1b). The phase change parameters of eicosane, PEG4000 and the composites are listed in Table 1.

### Microstructure characteristics

Notably, the rGO scaffold can absorb more than 97% eicosane or PEG4000 without leakage during phase change owing to its strong intermolecular interaction (hydrogen bonds or C-H…π) and capillarity (Table 1)^36^. The rGO sheets reveal 3D interconnected porous network and high porosity (Figure 1c and Figure S2). The cross-sectional scanning electron microscope (SEM) images of eicosane-infiltrated end of the composite exhibit that eicosane wraps the rGO sheets and fills in the intersheet spaces (Figure 1d and Figure S3). Since the encapsulated eicosane in aerogel can be easily melted by the focused beam of electrons owing to its low melting point (35°C), the phase transition process was observed during SEM image measurement. Unlike the eicosane, the solid PEG4000 discretely disperses on intersheets of rGO aerogel as white nanoparticles rather than thin films attached on the sheets (Figure 1e). This should be attributed to the higher crystallinity and a faster rate of crystallization than those of eicosane. Moreover, it maintains solid-state even if exposed to the strong electron beam of SEM owing to its higher phase transition temperature (60°C) in the other end of the aerogel scaffold. Since the immiscible nature of eicosane and PEG4000 prevents their mutual diffusion as shown in Figure 1f, there is a clear boundary between the two phases. Therefore, the binary phase change composites can present a promising recycling performance during phase transition.

### Thermal properties

**Figure 2:**
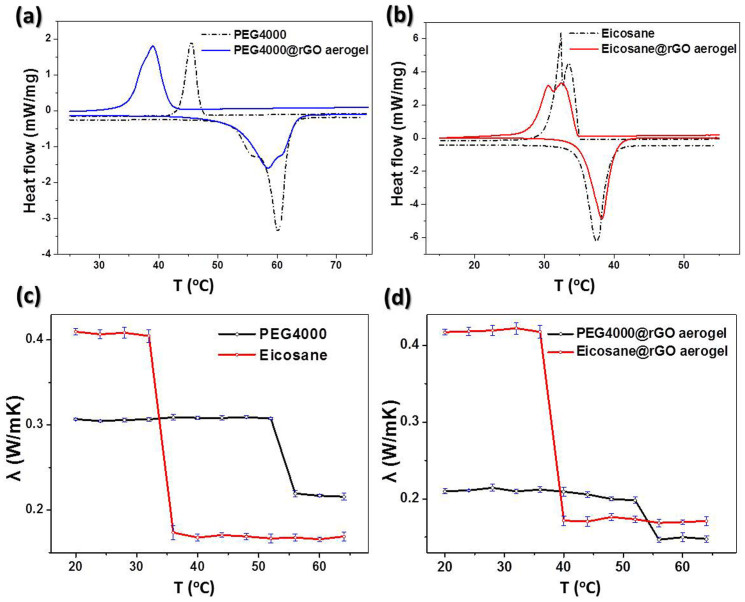
Thermal properties of PCM@rGO aerogel composites. (a) DSC curves of PEG4000 and composite with PEG4000 weight percentage of 97.8%. (b) DSC curves of eicosane and composite with eicosane weight percentage of 97.5%. (c) Thermal conductivities of pure PCMs, recorded across a temperature range of 20 to 64°C. (d) Thermal conductivities of two composites, recorded across a temperature range of 20 to 64°C.

Thermal properties were investigated by Differential Scanning Calorimetry (DSC) and thermal conductivity measurements. Interestingly, the solid-liquid phase transition point of PEG4000@rGO is 6.7°C lower than that of pure PEG4000 (Figure 2a and Table 1), which should be attributed to the fact that the porous rGO aerogel partly interrupts the inherent intermolecular hydrogen bonds and eventuates in a lower crystallinity of PEG4000, while the weak polarity of rGO leads to a weak binding interaction with PEG molecules, and therefore a remarkable phase transition and an enthalpy depression happen^36^. On the contrary, the eicosane@rGO composite displays a slightly higher phase transition temperature and lower enthalpy depression than those of pure eicosane (Figure 2b and Table 1). It indicates that the crystallinity of eicosane molecules within rGO aerogel has no significant change and the intermolecular interactions between rGO layer and highly dispersed eicosane film have compensated enthalpy depression of eicosane^29^.

The thermal conductivities of all the samples were measured in a temperature range from 20 to 64°C via hot wire method, and the error bars were the relative standard deviation (RSD) and the details were listed in Table S1–S4. The thermal conductivities of eicosane and PEG4000 are 0.409 and 0.308 W·m^−1^·K^−1^ in solid state and rapidly drop to 0.170 and 0.216 W·m^−1^·K^−1^ after solid-to-liquid phase transition, respectively^37^. Owing to high porosity, the rGO scaffold was thermal insulator with the thermal conductivity of only 0.0323 W·m^−1^·K^−1^, which is 10 times lower than that of the solid states of two pure PCMs. Below the phase transition temperature of eicosane (36°C), the thermal conductivity of eicosane@rGO composite was close to the pure eicosane. Similarly, the thermal conductivity of eicosane@rGO has an obvious saltation when the temperature is higher than 36°C, implying the PCMs act the vital role to determine the thermal conductivity of their composites. Notedly, PEG4000 was still in the solid-state during the phase transition of eicosane with a stable thermal conductivity value because of its higher melting point (Figure 2c). According to the SEM images, the solid PEG4000 discretely disperses on intersheets of rGO aerogel (Figure 1d and 1e), which results in a lower thermal conductivity of PEG4000@rGO composites for both solid and liquid states than those of pure PEG4000 since the super-low thermal conductivity of rGO skeleton.

### Thermal Rectification

**Figure 3:**
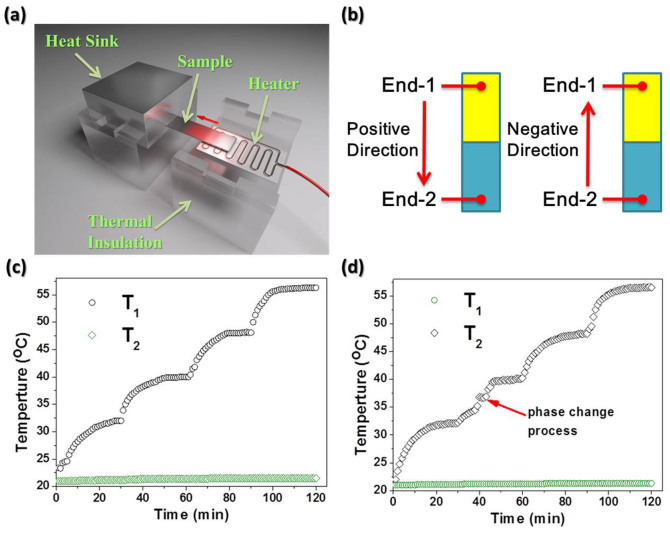
Experimental results of the thermal rectifier. (a) Schematic of the experimental setup to measure thermal rectification. (b) The direction of heat flux. Monitored temperatures as a function of time of positive direction (c) and negative direction (d) at different heater temperatures.

The phenomenon of thermal rectification can be demonstrated on binary eicosane/PEG4000 stuffed rGO aerogels. One end with PEG4000 is denoted as End-1, and the other with eicosane as End-2. The sample was adhered to a heater and a heat sink, and then was placed on thermal insulation (Figure 3a). The positive thermal bias direction is defined as the heat flux from End-1 to End-2 (from the heater to the heat sink), as illustrated in Figure 3b. On the contrary, when the temperature of End-2 is higher than that of End-1, the thermal bias is denoted as negative. The heat power (*P*) was to keep the stationary state of heat flux, which can be calculated according to Eq. (1) described by
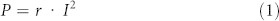
Where *r* and *I* are the electrical resistance of the heater and electrical current flowing through the heater. The calculated results of heat powers are different from the positive (*P_+_*) and negative (*P_−_*) direction at the same temperature bias, which show heat rectification effect in the composite. The thermal rectification coefficient *R* was defined as shown in Eq. (2), described by
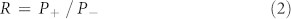
There is no thermal rectification phenomenon in such a case of *R* = 1.0. The larger the R value (*R* > 1.0), the higher the thermal rectification efficiency. We set four programmed temperature for the hot side, 32.0, 40.0, 48.2 and 56.3°C, respectively, as illustrated in Figure 3c and Table S5. The cold side kept constant temperature (ca. 21°C, T_2_ in Figure 3c, 3d) during the tests. The magnitude of heat flux along the positive direction was the same as that along the negative one. When put the End-1 at the hot side, both ends were still in the solid-state (positive direction) with increasing temperature of hot side to 40.0°C which is higher than the solid-to-liquid phase transition temperature of eicosane. However, when End-2 was at hot side, the solid-liquid phase change of eicosane occurred in this end (negative direction), which has been pointed out in Figure 3d. In this case, the heat power of positive direction (*P_+_*) was larger than that of the negative (*P_−_*) resulted in the value *R* of 1.10, implying the thermal rectification. Kept elevating the temperature to 48.2°C, the *R* value can rise to 1.23 where almost all eicosane has melted within the aerogel. With the increase of temperature (*e.g.* 56.3°C), part of solid-to-liquid phase transition of eicosane near the boundary point at positive heat flux direction leads to the local thermal conductivity change, and therefore declines the value of *P_+_/P_−_*.

**Figure 4:**
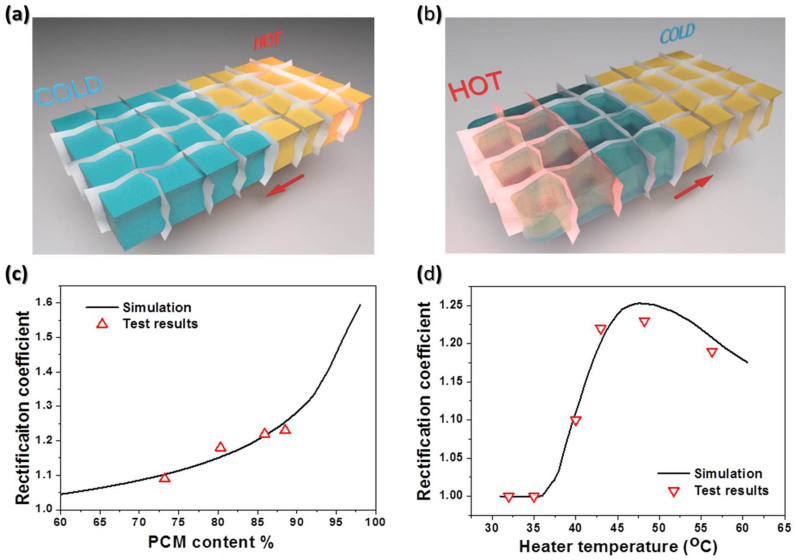
Simulation results of thermal rectifier by FEM. Schematic structure of the simulation model by FEM method, positive direction (a) and negative direction (b). The left part represented eicosane and the right was PEG4000. The arrow shown the direction of heat flux, and we heated the rectifier at the red side. (c) Thermal rectification coefficient with different proportion of PCMs. (d) Thermal rectification coefficient with different heater temperatures.

To further investigate the PCM-based thermal rectifier, a series of samples with different proportion of PCM were tested. It shows the positive relation between the proportion of PCM and thermal rectification coefficient *R*, as illustrated in Figure 4c and Table S6. In addition, because the aerogel was infiltrated asymmetrically by different PCMs, the cycling property was investigated. After five cycles at 48.2°C, the coefficient of rectifier was still 1.22 (Table S7), indicating a stable performance during the repeating rectification process.

To understand the underlying mechanism of the rectification phenomenon, we perform finite element modeling (FEM) simulation to study the heat flux and temperature distribution in the system (Figure 4a, b). As is well known, heat could be transferred by thermal transmission, natural convection and radiation. In our system, heat was mainly transferred by sample sheet since heat isolation experimental setup, the radiation power could be calculated according to Stepan-Boltzmann's law through Eq. (3) described by
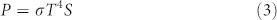
where value of Stepan constant *σ* was 5.67 × 10^−8^ W·m^−2^·K^−4^, and T, S were the absolute temperature and the surface area of the rGO aerogel sheet. The natural convection was negligible since our experiment was carried out in a vacuum space.

In FEM simulation, the rectifier composition was treated as rGO sheet framework with inner space filled with PCMs. The thermal conductivity of rGO aerogel was 0.0323 W·m^−1^·K^−1^ and the thermal conductivities of PCMs were shown in Figure 2d. Heat powers *P_+_* and *P_−_* were calculated at the same temperature bias by FEM.

The thermal rectification coefficient with different ratio of PCMs was shown in Figure 4c. The mechanism of thermal rectification was based on the thermal conductivity saltation of PCM at phase transition temperature. The coefficient depended on the proportion of heat flux in PCM. As a result, the simulated coefficient was in proportion to the content of PCMs (the curve in Figure 4c), which coincided with the experimental measurement result. So the coefficient could be tailored via infiltration of different content of PCMs.

Furthermore, the heater temperature had greatly influenced on the thermal rectification. The simulated curve of the relationship between heater temperature and rectification coefficient was illustrated in Figure 4d. When the temperature was lower than 36°C, the rectification coefficient was a constant of 1.0, which suggested that the heat flux along the positive direction was the same as that along the negative direction. When eicosane began to melt, the coefficient increased with the rising of the heater temperature and achieved the peak value about 48°C. The phase transition of lower temperature end acts as a switch, which could turn on the thermal rectification. The rectification coefficient starts to decline when the temperature continues to rise, because the partial liquefaction of eicosane in cold side under positive thermal bias leads to the reduction of difference in heat flux between positive and negative direction. Six experimental coefficients were acquired at different heater temperatures. The experimental results were in good agreement with the simulated as shown in Figure 4d and Table S8. So the thermal rectifier could be activated by means of solid-to-liquid phase transition and modulated by different heater temperatures.

**Figure 5:**
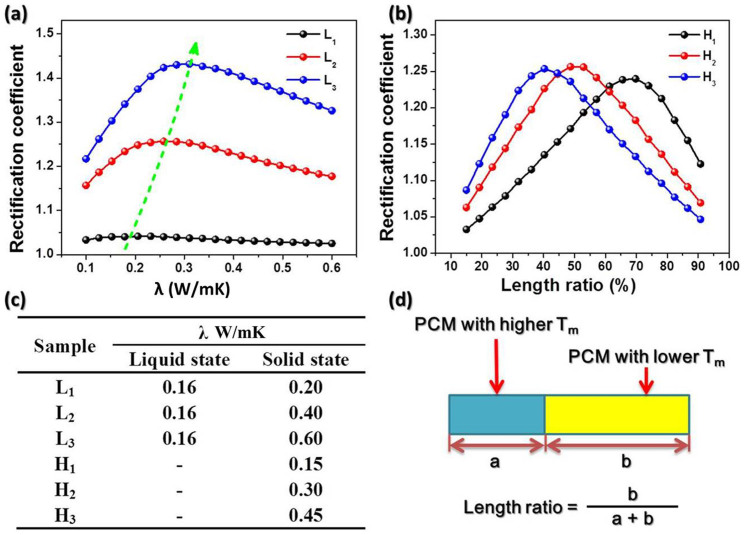
(a) Simulation result of thermal rectification coefficient at different thermal conductivity of PCMs. The three lines (L_1_ ~ L_3_) represent different thermal conductivities of PCM with a low melting point, and PCM content is 88.3%. (b) Simulation result of the relationship between rectification coefficient and length ratio of PCM with low melting point. Its thermal conductivity is 0.4 and 0.16 W/mK in solid and liquid state, and PCM content is 88.3%. Three lines ((H_1_ ~ H_3_) denote different thermal conductivities of PCM with high melting point. (c) The thermal conductivity of different PCM for simulation. (d) Schematic of the length ratio. Three lines (D, E, and F) denote different thermal conductivities (0.15, 0.30, and 0.45 W/mK, respectively) of PCM in high melting point PCM end.

Besides the results of experiment and simulation above, we have considered other influences on thermal rectification, such as thermal conductivity of PCMs and loading ratio of different PCMs. In Figure 5a, the three lines represent different thermal conductivities of PCM with a lower melting point. The thermal conductivity of L_1_, L_2_ and L_3_ are listed in Figure 5c with PCM content of 88.3%. According to the simulated curves, the larger the thermal conductivity variation between solid and liquid states of PCM in low melting point end, the higher rectification coefficient is achieved. Furthermore, the conductivity of PCM with higher melting point can also contribute to the rectification. As the arrow illustrated in the Figure 5a, the promising blocks are displayed on the curves.

Figure 5b reveals the relationship between rectification coefficient and length ratio of PCM in the lower melting point end. Simulation parameters were set at 0.4 and 0.16 W·m^−1^·K^−1^ in solid and liquid state for low melting point PCM end. Three lines (H_1_ ~ H_3_) denote different thermal conductivities (0.15, 0.30, and 0.45 W·m^−1^·K^−1^, respectively) of PCM in high melting point end. On the basis of simulation results in Figure 5b, each curve has the best value of coefficient with different length ratio of PCM. In order to obtain optimal coefficient, with the lower thermal conductivity of PCM in high melting PCM end, more PCM in low melting end should be infiltrated. The best length ratio in our simulation was nearly 50% (line H_2_), which matches well with our experimental results.

## Discussion

Thermal rectification has been studied in different systems. Different mechanisms are responsible for thermal rectification, such as geometry, materials, surface preparation and so on. In this work, we invent a novel method to construct the temperature-responded rectifier by using thermal conductivity saltation of organic solid-liquid phase transition. In general, the thermal conductivity of the solid-state of materials is higher than its liquid state because the phonon is easier to transfer in the solid-state. The thermal conductivity variations of different substances during the solid-liquid phase transition are not entirely the same. As examples shown in Figure S6, lauric acids, methyl stearate and eicosane have the similar phase-transition temperature falling into 32–42°C. However, their thermal conductivity variations are quite different during phase transition, in which eicosane shows the highest amplitude of variation. Furthermore, organic PCM with the similar polarity can be mutually miscible by either polar-polar or nonpolar-to-nonpolar interactions. Eicosane is a typical hydrocarbon with weak polarity, which is quite different from alcohol or carboxyl-based PCMs like PEGs. Therefore, we have selected eicosane/PEG couple to avoid their mutual solubility. On the other hand, nano-confinement of solid-to-liquid PCMs within porous materials can not only prevent leakage of PCM during phase transition, but also realize the function of PCMs. For this conception, we tailored rGO aerogel as the skeleton to encapsulate binary eicosane/PEG in virtue of its high porosity and the amphipathic nature. Most importantly, the rGO aerogel with superlow thermal conductivity never greatly affects the thermal conductivity variation of host PCMs during phase transition.

**Figure 6:**
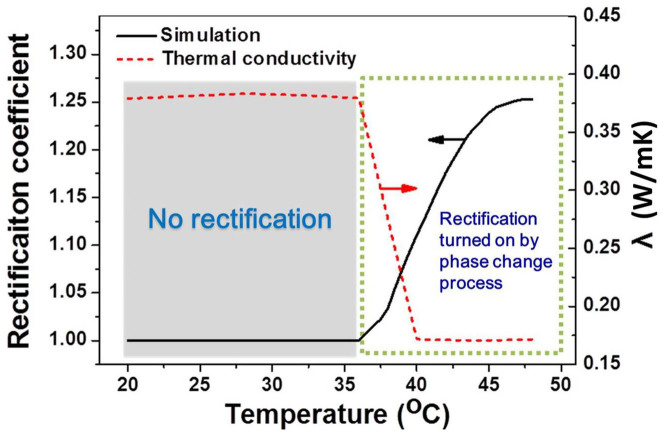
Thermal rectification switched on by the phase transition process.

As shown in Figure 6, when the temperature is lower than the melting point of PCM (low temperature end), there is no thermal rectification. Because of the sharply fall of thermal conductivity at the melting point^34^, phase change presents as a switch which can turn on the thermal rectifier. Recently, a thermal rectifier was constructed by using MnV_2_O_4_ and La_1.98_Nd_0.02_CuO_4_^25^, in which the structural phase transition driven by the orbital ordering in MnV_2_O_4_ induces thermal conductivity jump at 57 K, and resulted in thermal rectification effect in the system. As phase transition temperature of MnV_2_O_4_ is 57 K, this thermal rectifier can only work under low temperature. In contrast, the thermal rectifier realized in the present work is based on binary phase change composites. The present thermal rectifier can work around room temperature, and the range of working temperature is controllable. Hence, the present work not only demonstrated a viable route to construct a thermal rectifier, but also provided a general design principle for controlling the working temperature.

In summary, we have demonstrated a novel strategy to fabricate a temperature-controllable thermal rectifier by employing phase change composites. The thermal rectification can be modulated through selecting PCMs with specific melting temperature, or tuning the loading of phase change materials. Phase transition in porous networks instantaneously results in the thermal conductivity saltation in one end, and therefore switching on or off the thermal rectification. The stated technology has broad applications for thermal energy control in macroscopic scale such as energy-efficiency building or nanodevice temperature management. The generality of the approach is also demonstrated for multicomponent phase change composites with different temperature gradients.

## Methods

### Sample preparation

Graphene oxide (GO) used in this case was synthesized according to the references 31, 32. 0.4 g Vitamin C was added into a 100 mL beaker containing 60 mL 5.0 mg/mL GO aqueous solution with vigorous magnetic stirring until VC dissolved completely. The rGO hydrogel was prepared in the Petri dish at 45°C for 12 h. After CO_2_ supercritical dry, the rGO aerogel was obtained. The resultant aerogel was cut into rectangular blocks, which will be infiltrated by PEG4000 and eicosane. In order to get the samples with various contents, we used alcohol to dilute the PCMs. The aerogel sheets were infiltrated by the solutions with different concentrations, and dried them in the oven. After weighing, the samples with various contents of PCMs have been prepared.

### Microstructure analysis

The SEM images were performed to characterize the microstructure using a field-emission microscope (Hitachi-s4800) operated at 10 kV.

### DSC and TG measurements

The DSC data were obtained using a Setaram DSC 131 evo within an Al 30 μL pan. All the sample weights are between 1 and 5 mg, and the temperature change rate is 5°C/min under Ar protection. The thermogravimetric (TG) curves were obtained with a TASDT-Q600 Thermogravimetric Analyzer. The weights of samples were between 1 and 5 mg and the heating rate is 10°C/min under N_2_ atmosphere. Thermal conductivity was carried out with hot-wire thermal conductivity instrument (Xiatech TC3010), and a circulating oil bath was used to adjust the temperature.

### Thermal conductivity measurement

Thermal conductivity was characterized by using hot wire method. Before measurement, the detector of hot wire is closely clamped with two reticular samples. The temperature of samples and detector was monitored by using a programmed software. The measurement would not start until the fluctuation rate of temperature is less than ±50 mK/5 min. Every measurement is tested 5 times at a giving temperature. Two parallel measurements should have an interval of 10 minutes to ensure that the inner part of sample can reach the equilibrium state of the measured temperature.

### Thermal rectification test

The sample was adhered to a heater and a heat sink, then it was placed on thermal insulation (Fig. 3a). Joule heat generated by heater flux was transferred from the heater to the heat sink with a temperature gradient. The heat was absorbed by the heat sink, so the temperature of this side could keep constant. Thermal conductance could be calculated from heat flux and temperature difference.

### Simulations

Our simulations are based on a model that the thermal rectifier is in contact with two heat baths at the two ends. The size of thermal rectifier is 25.0 × 6.0 × 0.8 mm^3^. Upon electrical current flow through the heater, Joule heating can create a high temperature side. We only consider the steady-state case, thus 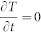. Natural convection and radiation as the heat transfer mechanism between the system and the surrounding air are included, as adopted in References 38, 39. The finite element modelling is used to perform a steady-state thermal analysis to evaluate temperature profile. We employed COMSOL MULTIPHYSICS by COMSOL, a finite element program, in our simulations.

## Author Contributions

R.C. and Y.C. contributed equally to this work. R.C. and R.Z. designed the experiments. R.C., R.Y. and Z.L. prepared samples, performed the SEM, TGA, DSC, and thermal conductivity measurements, and analyzed data. H.T., Y.C. and T.R. designed the equipment of thermal conversion. Y.C., Y.S., C.L., Y.Y. and H.T. performed the simulation and rectification test. R.C., Y.C., R.Y., R.Z. and G.Z. co-wrote the article. All authors contributed to results and discussion.

## Supplementary Material

Supplementary Information Controllable Thermal Rectification Realized in Binary Phase Change Composites

## Figures and Tables

**Table 1:** Latent heat and content values of pure PCMs and the composites

Sample	Latent Heat J/g	Melting Point °C	PCM content %
PEG4000	177.6	60.1	100.0
PEG4000@rGO aerogel	143.5	56.8	97.2
Eicosane	244.1	35.4	100.0
Eicosane@rGO aerogel	213.8	36.3	97.5
