# Supplementary material for: Controllable Thermal Rectification Realized in Binary Phase Change Composites
Source: Sci Rep. 2015 Mar 9;5:8884. doi: 10.1038/srep08884 (PMC4352864; doi:10.1038/srep08884)
Supplement: Supplementary Information — Controllable Thermal Rectification Realized in Binary Phase Change Composites [file srep08884-s1.doc]

**Controllable Thermal Rectification Realized in Binary Phase Change Composites**

Renjie Chen,^1,ǂ^ Yalong Cui,^2,3,ǂ^ He Tian,^2,3^ Ruimin Yao,^1^ Zhengpu Liu,^1^ Yi Shu,^2,3^ Cheng Li,^2,3^ Yi Yang,^2,3^Tianling Ren,^2,3,*^ Gang Zhang,^4,*^ Ruqiang Zou^1,*^

^1^ *Department of Materials Science and Engineering, College of Engineering, Peking University, Beijing 100871, China. ^2^ Institute of Microelectronics, Tsinghua University, Beijing 100084, China. ^3^ Tsinghua National Laboratory for Information Science and Technology (TNList), Tsinghua University, Beijing 100084, China. ^4^ Institute of High Performance Computing, Singapore 138632, Singapore. Correspondence and requests for materials should be addressed to T.R. (email: rentl@tsinghua.edu.cn), G.Z. (e-mail: zhangg@ihpc.a-star.edu.sg), and R. Z. (e-mail: rzou@pku.edu.cn).*

^ǂ^ These authors contributed equally to this work.

GO used in this work was synthesized as follows [1]: Concentrated H_2_SO_4_ (25mL) in a 100mL beaker was heated to 90°C. K_2_S_2_O_8_ (5.0g) and P_2_O_5_ (5.0g) were added in sequence with continuous stirring until the reactants were completely dissolved. The mixture was then cooled down to 80°C. Graphite powder (6.0g) was then added to above mixture. The resulting mixture was kept at 80 °C for 4.5h, after which the mixture was diluted with 1 L of distilled water and left overnight. The mixture was then filtered and washed to remove all soluble substances, and the corresponding pretreated graphite was transferred to a drying dish and left overnight under ambient conditions. Next, concentrated H_2_SO_4_ (230mL) was put into a 1 L Erlenmeyer flask and then cooled down in an ice bath. The above-mentioned pretreated graphite and KMnO_4_ (30.0g) were added slowly in sequence and then magnetically stirred for ca. 10 min. The mixture was then allowed to react at 35 °C for 2h, after that, distilled water (460mL) was slowly added. When the temperature of the mixture remained constant, another 1.4 L of distilled water was added to the mixture. The mixture was stirred for another 2 h, thereafter 25mL of 30% H_2_O_2_ was added to the mixture. The mixture was allowed to stand for at least 12h then the clear supernatant was decanted. The remaining precipitate was washed with a large volume of 5% HCl solution followed by distilled water to remove impurities. The final solution was concentrated to 10 mg/mL.


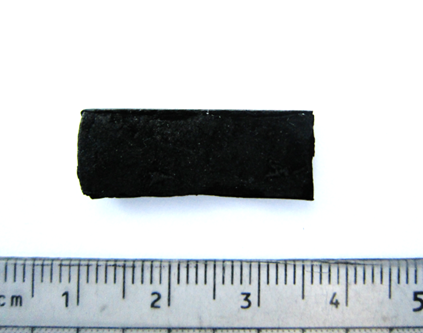


Fig. S1 Photo of sample sheet.


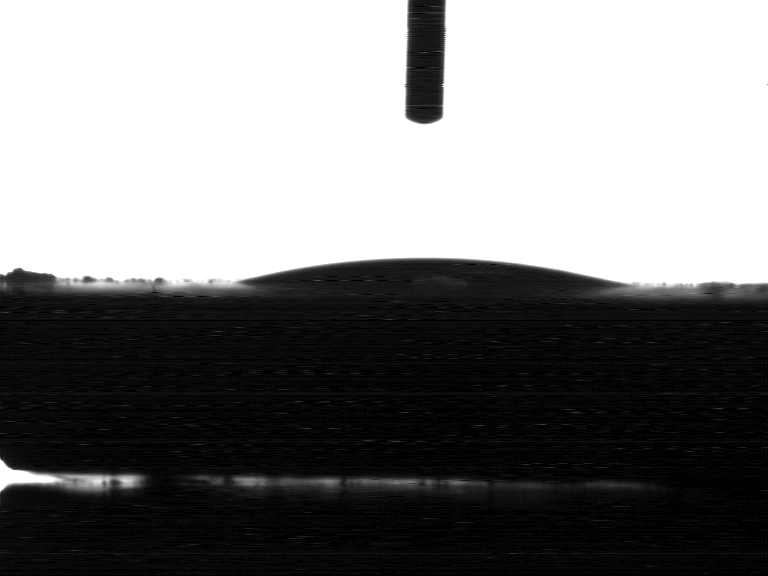


(a)


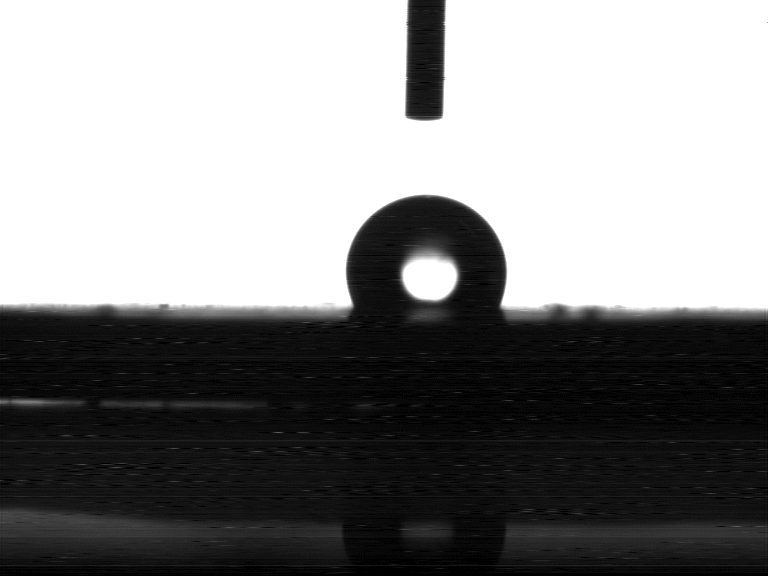


(b)

Fig. S2 The contact angle measuring for PEG4000(a) and Eicosane(b).


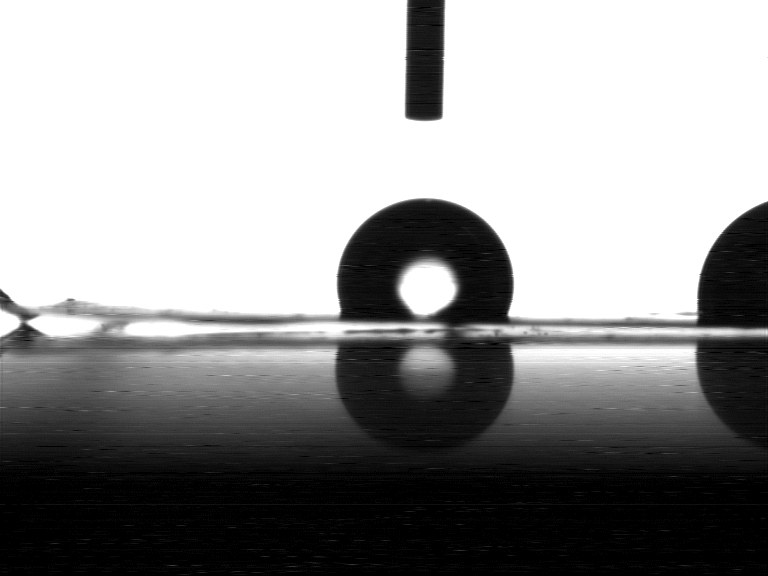


(a)


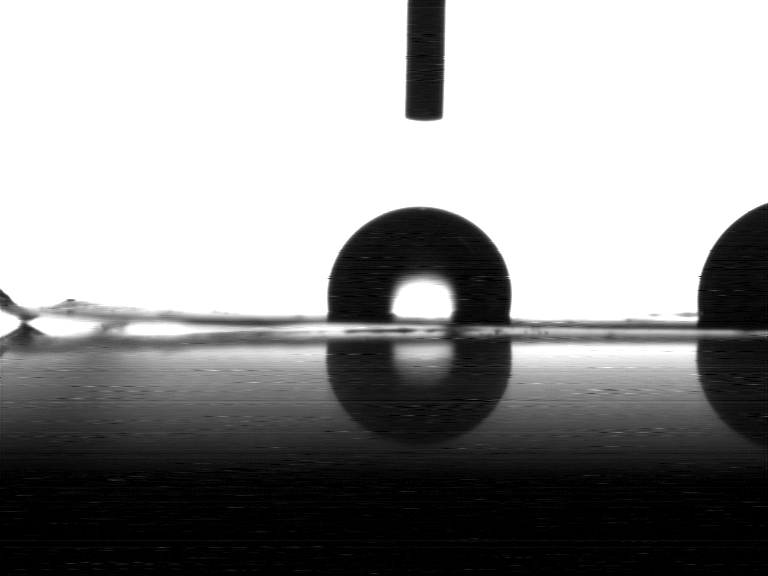


(b)

Fig. S3 The contact angle measuring for rGO aerogel.

The contact angle measuring pictures were obtained by Dataphysics OCA20 Contact Angle Measuring System. We can’t get the accurate data of the rGO aerogel and composites, because the samples could infiltrate the solvent quickly due to their porous structure. So what we can apply are the contact angle pictures of two pure PCMs, and the contact angle of PEG4000 and Eicosane are 18.3^o^ and 116.1^o^, respectively (water was the test solvent). According to Fig. S8, we can find that the water could infiltrate into the aerogel by capillary pressure, so the contact angle wasn’t a constant. The angle is change from 109.4^o^ to 90.3^o^.


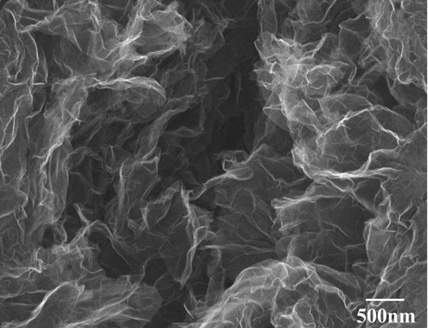


Fig. S4 SEM image of rGO aerogel


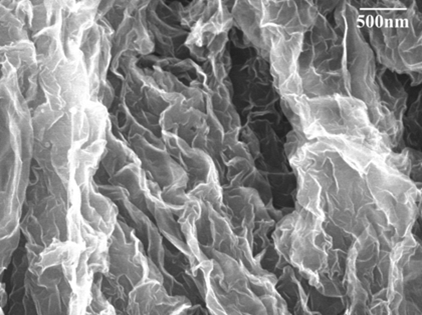


(a)


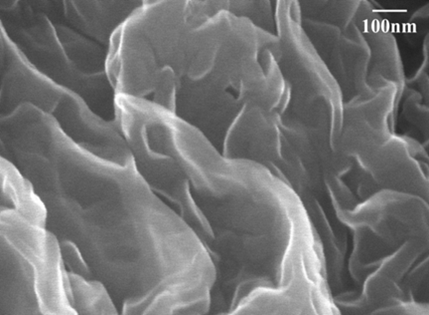


(b)

Fig. S5 SEM images of eicosane@rGO aerogel


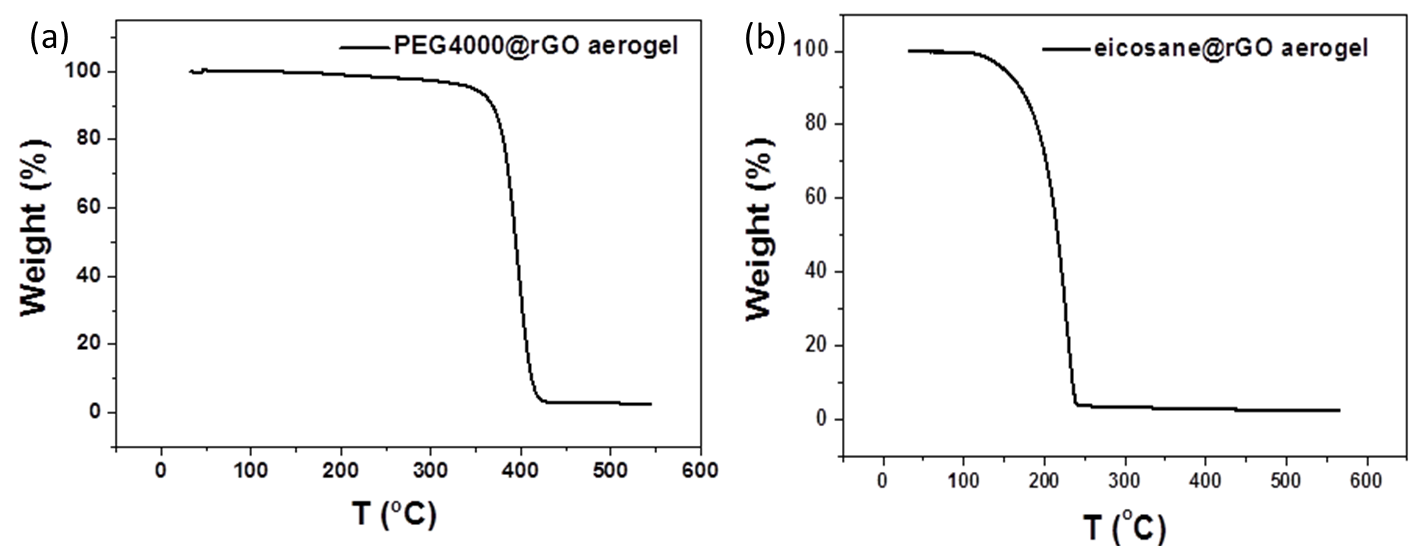


Fig. S6 TGA curves of the DSC samples, (a)PEG4000@rGO aerogel, (b)eicosane@rGO aerogel


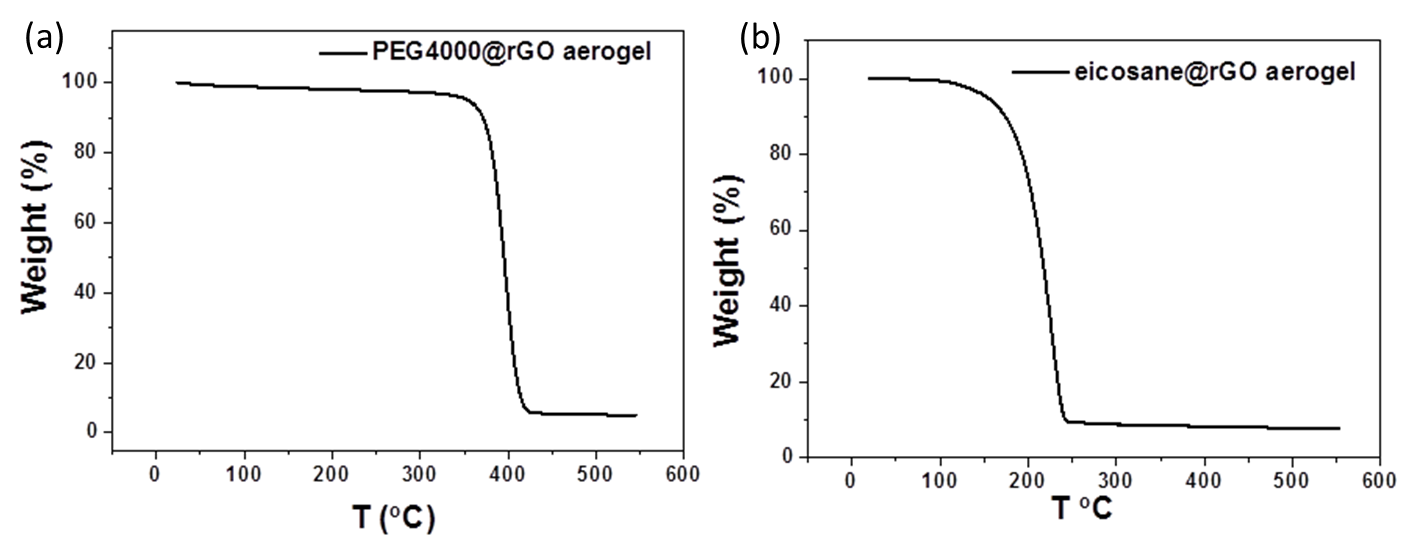


Fig. S7 TGA curves of the composites used for the thermal conductivity tests, (a)PEG4000@rGO aerogel, (b)eicosane@rGO aerogel


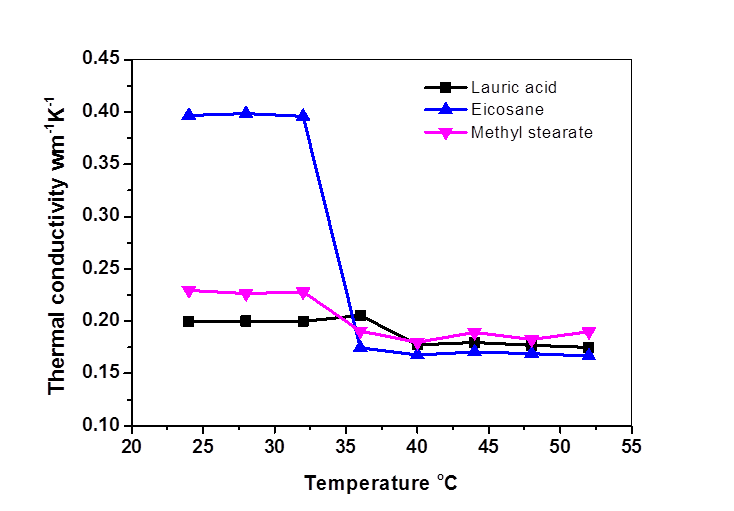


Fig. S8 The temperature-dependent thermal conductivity of different PCMs.

The error bars for thermal conductivity measurement are actually the relative standard deviation (RSD), which is determined by the Equation:

$$\sigma=\sqrt{\frac{1}{N-1}\sum_{i=1}^{N} (x_{i}-\mu)^{2}}$$

where “*σ*” is the RSD value, “*N*” means the total number of values, “*x_i_*” is the measured thermal conductivity value, “*µ*” is the average thermal conductivity value. The specific measured values and the RSD values are listed below, which proves our RSD values are in the small range of 10^-3^ W•m^-1^•K^-1^:

Table S1 the specific measured values and the RSD values of PEG4000

| T / ^o^C | K1 | K2 W•m^-1^•K^-1^ | K3 W•m^-1^•K^-1^ | K4 W•m^-1^•K^-1^ | K5 W•m^-1^•K^-1^ | K(average) W•m^-1^•K^-1^ | RSD W•m^-1^•K^-1^ |
| --- | --- | --- | --- | --- | --- | --- | --- |
|  | W•m^-1^•K^-1^ |  |  |  |  |  |  |
| 20.05 | 0.3080 | 0.3049 | 0.3071 | 0.3074 | 0.3067 | 0.3068 | 0.00117 |
| 24.15 | 0.3153 | 0.3157 | 0.3135 | 0.3145 | 0.3159 | 0.315 | 0.00099 |
| 27.90 | 0.3081 | 0.3093 | 0.3138 | 0.313 | 0.3105 | 0.3109 | 0.00242 |
| 32.00 | 0.3097 | 0.3055 | 0.3045 | 0.3076 | 0.3061 | 0.3067 | 0.00203 |
| 35.95 | 0.3031 | 0.3101 | 0.3149 | 0.3137 | 0.3042 | 0.3092 | 0.00538 |
| 40.10 | 0.3097 | 0.3067 | 0.3072 | 0.3088 | 0.3109 | 0.3087 | 0.00174 |
| 44.10 | 0.3110 | 0.3061 | 0.3063 | 0.3115 | 0.3082 | 0.3086 | 0.00254 |
| 48.05 | 0.3097 | 0.3094 | 0.307 | 0.3093 | 0.3114 | 0.3094 | 0.00157 |
| 52.15 | 0.3061 | 0.3077 | 0.3081 | 0.3079 | 0.3094 | 0.3078 | 0.00118 |
| 55.85 | 0.2246 | 0.2173 | 0.2213 | 0.2151 | 0.2188 | 0.2194 | 0.00367 |
| 60.10 | 0.2145 | 0.2192 | 0.2171 | 0.2169 | 0.2168 | 0.2169 | 0.00167 |
| 64.05 | 0.2166 | 0.2172 | 0.2101 | 0.2212 | 0.2144 | 0.2159 | 0.00407 |
| 67.90 | 0.2143 | 0.2163 | 0.2124 | 0.2141 | 0.2131 | 0.2140 | 0.00148 |

Table S2 the specific measured values and the RSD values of eicosane

| T / ^o^C | K1 | K2 W•m^-1^•K^-1^ | K3 W•m^-1^•K^-1^ | K4 W•m^-1^•K^-1^ | K5 W•m^-1^•K^-1^ | K(average) W•m^-1^•K^-1^ | RSD W•m^-1^•K^-1^ |
| --- | --- | --- | --- | --- | --- | --- | --- |
|  | W•m^-1^•K^-1^ |  |  |  |  |  |  |
| 20.15 | 0.4151 | 0.4101 | 0.4052 | 0.4088 | 0.4091 | 0.4097 | 0.00356 |
| 23.80 | 0.4038 | 0.4009 | 0.4072 | 0.4157 | 0.4061 | 0.4067 | 0.00556 |
| 28.10 | 0.4093 | 0.4044 | 0.4051 | 0.3987 | 0.4158 | 0.4067 | 0.00635 |
| 32.10 | 0.4133 | 0.3973 | 0.4019 | 0.3995 | 0.4127 | 0.4049 | 0.00754 |
| 36.05 | 0.1691 | 0.1639 | 0.1795 | 0.1716 | 0.1846 | 0.1737 | 0.00828 |
| 39.90 | 0.1668 | 0.1701 | 0.1621 | 0.1659 | 0.1733 | 0.1676 | 0.00426 |
| 43.95 | 0.1702 | 0.1749 | 0.1677 | 0.1696 | 0.1709 | 0.1707 | 0.00265 |
| 48.10 | 0.1708 | 0.164 | 0.1689 | 0.1742 | 0.1669 | 0.169 | 0.00386 |
| 52.15 | 0.1607 | 0.1634 | 0.1692 | 0.1669 | 0.1744 | 0.1669 | 0.00530 |
| 55.85 | 0.1700 | 0.1607 | 0.1663 | 0.1718 | 0.1661 | 0.167 | 0.00427 |
| 60.05 | 0.1666 | 0.1668 | 0.1616 | 0.1656 | 0.1687 | 0.1659 | 0.00263 |
| 64.10 | 0.1746 | 0.1699 | 0.1637 | 0.1685 | 0.1618 | 0.1677 | 0.00510 |

Table S3 the specific measured values and the RSD values of PEG4000@rGO-gel

| T / ^o^C | K1 | K2 W•m^-1^•K^-1^ | K3 W•m^-1^•K^-1^ | K4 W•m^-1^•K^-1^ | K5 W•m^-1^•K^-1^ | K(average) W•m^-1^•K^-1^ | RSD W•m^-1^•K^-1^ |
| --- | --- | --- | --- | --- | --- | --- | --- |
|  | W•m^-1^•K^-1^ |  |  |  |  |  |  |
| 16.20 | 0.2147 | 0.2076 | 0.2185 | 0.2144 | 0.2061 | 0.2123 | 0.00522 |
| 20.05 | 0.2088 | 0.2132 | 0.2111 | 0.2103 | 0.2047 | 0.2096 | 0.00318 |
| 24.10 | 0.2103 | 0.2098 | 0.2110 | 0.2112 | 0.2134 | 0.2111 | 0.00138 |
| 28.05 | 0.2139 | 0.2202 | 0.2154 | 0.2165 | 0.2076 | 0.2147 | 0.00461 |
| 31.90 | 0.2104 | 0.2057 | 0.2092 | 0.2144 | 0.2081 | 0.2096 | 0.00321 |
| 36.10 | 0.2082 | 0.2116 | 0.2095 | 0.2127 | 0.2183 | 0.2121 | 0.00391 |
| 40.15 | 0.2143 | 0.2095 | 0.219 | 0.2061 | 0.2039 | 0.2106 | 0.00614 |
| 44.05 | 0.2057 | 0.2036 | 0.2048 | 0.2019 | 0.2115 | 0.2055 | 0.00364 |
| 48.05 | 0.2036 | 0.2014 | 0.1989 | 0.1967 | 0.1985 | 0.1998 | 0.00270 |
| 51.80 | 0.1998 | 0.2073 | 0.1956 | 0.1988 | 0.1976 | 0.1998 | 0.00446 |
| 56.10 | 0.1438 | 0.1441 | 0.1457 | 0.1496 | 0.1524 | 0.1471 | 0.00375 |
| 60.15 | 0.1531 | 0.1455 | 0.1601 | 0.1479 | 0.1483 | 0.1510 | 0.00580 |
| 63.95 | 0.1405 | 0.1482 | 0.1523 | 0.1499 | 0.1507 | 0.1483 | 0.00461 |
| 68.25 | 0.1430 | 0.1563 | 0.1424 | 0.1415 | 0.1531 | 0.1473 | 0.00691 |

Table S4 the specific measured values and the RSD values of eicosane@rGO-gel

| T / ^o^C | K1 | K2 W•m^-1^•K^-1^ | K3 W•m^-1^•K^-1^ | K4 W•m^-1^•K^-1^ | K5 W•m^-1^•K^-1^ | K(average) W•m^-1^•K^-1^ | RSD W•m^-1^•K^-1^ |
| --- | --- | --- | --- | --- | --- | --- | --- |
|  | W•m^-1^•K^-1^ |  |  |  |  |  |  |
| 19.95 | 0.4221 | 0.4207 | 0.4129 | 0.4166 | 0.4152 | 0.4175 | 0.00383 |
| 24.05 | 0.4200 | 0.4254 | 0.4192 | 0.4101 | 0.4167 | 0.4183 | 0.00557 |
| 28.20 | 0.4205 | 0.4216 | 0.4087 | 0.4193 | 0.4262 | 0.4193 | 0.00646 |
| 32.10 | 0.4337 | 0.423 | 0.4222 | 0.4172 | 0.4129 | 0.4218 | 0.00780 |
| 35.80 | 0.4129 | 0.4158 | 0.4083 | 0.4197 | 0.4303 | 0.4174 | 0.00833 |
| 40.15 | 0.1703 | 0.1652 | 0.1715 | 0.1821 | 0.1705 | 0.1719 | 0.00620 |
| 44.20 | 0.1811 | 0.1725 | 0.1687 | 0.1639 | 0.1705 | 0.1713 | 0.00632 |
| 47.90 | 0.1662 | 0.1767 | 0.1758 | 0.1791 | 0.1774 | 0.175 | 0.00509 |
| 51.75 | 0.1728 | 0.1694 | 0.1706 | 0.1743 | 0.1804 | 0.1735 | 0.00430 |
| 56.15 | 0.1654 | 0.1625 | 0.1693 | 0.1766 | 0.1688 | 0.1685 | 0.00529 |
| 60.05 | 0.1667 | 0.1696 | 0.1706 | 0.1738 | 0.1679 | 0.1697 | 0.00273 |
| 64.20 | 0.1736 | 0.1609 | 0.1689 | 0.1765 | 0.1751 | 0.1710 | 0.00633 |
| 68.30 | 0.1643 | 0.1752 | 0.1624 | 0.1715 | 0.1731 | 0.1693 | 0.00563 |

Table S5 The results of the rectification test

| Test temperature ^o^C | *P_+_* mW | *P*- mW | Coefficient |
| --- | --- | --- | --- |
| 32.0 | 400 | 400 | 1.00 |
| 40.0 | 680 | 620 | 1.10 |
| 48.2 | 940 | 760 | 1.23 |
| 56.3 | 1120 | 940 | 1.19 |

Table S6 The results of the rectification test with different loading percentage

| Sample | PCM content% | Coefficient |
| --- | --- | --- |
| 1 | 88.5 | 1.23 |
| 2 | 85.9 | 1.22 |
| 3 | 80.3 | 1.18 |
| 4 | 73.2 | 1.09 |

Table S7 the results of cycling test

| Heater Temperature ^o^C | Cycling Num. | Ratio |
| --- | --- | --- |
| 48.2 | 1 | 1.23 |
| 48.2 | 5 | 1.22 |

Table S8 The results of the rectification test with different heater temperature

| Temperature ^o^C | Coefficient |
| --- | --- |
| 32.0 | 1.00 |
| 35.0 | 1.00 |
| 40.0 | 1.10 |
| 43.0 | 1.22 |
| 48.2 | 1.23 |
| 56.3 | 1.17 |

[1] Zhuyin Sui, Xuetong Zhang, Yu Lei, Yunjun Luo, Easy and green synthesis of reduced graphite oxide-based hydrogels, CARBON, 2011, 49, 4314-4321.
